# Intergenerational effects of parental positive childhood experiences on social skills development in Chinese preschoolers: the moderating role of the home-rearing environment

**DOI:** 10.3389/fpsyg.2025.1679531

**Published:** 2025-11-10

**Authors:** Xinyi Yuan, Zhu Zhu, Yanlin Wang, Ruifeng Zhao, Yixin Sun, Xiang Li, Haotian Gao, Tokie Anme

**Affiliations:** 1 International Community Care and Lifespan Development: Empowerment Sciences, Doctoral Program in Medical Sciences, University of Tsukuba, Tsukuba, Ibaraki, Japan; 2 School of Public Health and Nursing, Hangzhou Normal University, Hangzhou, Zhejiang Province, China; 3 College of Child Development and Education, Zhejiang Normal University, Hangzhou, China; 4 International Community Care and Lifespan Development: Empowerment Sciences, Faculty of Medicine, University of Tsukuba, Tsukuba, Ibaraki, Japan

**Keywords:** Chinese preschoolers, home-rearing environment, positive childhood experiences, social skills, intergenarational effects

## Abstract

**Background:**

While the detrimental intergenerational effects of adverse childhood experiences (ACEs) are well-documented, the potential developmental benefits associated with parental positive childhood experiences (PCEs) warrant further clarification, particularly concerning the social competence of offspring. The present study examines the relationship between parental PCEs and social skills in preschool-aged children, as well as the moderating role of the home-rearing environment.

**Methods:**

This cross-sectional study was conducted in three kindergartens in Xuzhou, Shangqiu, and Tianjin, representing the eastern and northern regions of mainland China. The study included 320 Chinese preschoolers (mean age = 4.76 years, SD = 1.01) and their parents. Parental PCEs were assessed using the Benevolent Childhood Experiences Scale, children’s social skills were measured with the Social Skills Scale, and the home-rearing environment was evaluated using the Index of Child Care Environment.

**Results:**

The findings demonstrated a significant positive association between parental PCEs and social skills in children (*β* = 1.15, *p* < 0.001). Furthermore, the quality of the home-rearing environment moderated this relationship, with more supportive environments amplifying the benefits of PCEs (*β* = 0.54, *p* < 0.05).

**Conclusion:**

These findings underscore the importance of incorporating both parental experiences and family environments in interventions aimed at fostering children’s social development.

## 1 Introduction

Social skills are defined as learned, socially appropriate behaviors that facilitate effective interpersonal interactions and reduce the likelihood of inappropriate responses (Gresham and Elliott, 1984), are critical for children’s future social adaptation and psychological well-being (Mohammadi et al., 2019; Siranchuk and Bondarenko, 2023). In early childhood, the development of these skills is shaped by a range of familial and environmental factors, with parental childhood experiences emerging as a significant influence through intergenerational transmission (Zhang et al., 2023; Zhu et al., 2023; Blackwell et al., 2024).

Parental childhood experiences, encompassing both adverse and positive events, play a pivotal role in shaping parenting behaviors and, consequently, child development. Adverse childhood experiences (ACEs) are potentially traumatic life events that occur before the age of 18, including child abuse (physical, emotional or sexual) and neglect, mental illness of family members, parental divorce or separation, and family member substance use (Felitti et al., 1998). Existing research has focused on ACEs, showing that mothers with more exposure to ACEs can pass the negative effects of ACEs to the next generation through poor health behaviors, parenting styles and broader family relationship health, which increases the risk of problem behavior in offspring (Greene et al., 2020; Narayan et al., 2021; Reese et al., 2022; Racine et al., 2023). At the social level, it is reflected in an increased risk of socioemotional difficulties and a delay in personal-social development in children (Zhang et al., 2023; Folger et al., 2018).

Positive Childhood Experiences (PCEs) refer to beneficial experiences before age 18 characterized by perceived safety, stable support systems, structured household routines, comforting belief systems, and strong community connections (Narayan et al., 2018). While the detrimental effects of ACEs have been widely documented, limited research has examined the potential protective impact of parental PCEs on the development of the child.

### 1.1 Parental PCEs and social skills development in young children

Parental PCEs are hypothesized to influence children’s development by shaping parents’ psychological well-being and parenting practices. Prior studies have shown that parental PCEs serve as protective factors for adult psychological well-being, reducing stress and enhancing emotional regulation (Crandall et al., 2019). These psychological benefits enable parents to engage in constructive parenting practices, and demonstrate positive parenting attitudes and behaviors, such as emotional warmth and responsive caregiving (Chen et al., 2008; Kosterman et al., 2011), which foster a home-rearing environment characterized by positive parent–child interactions (Haven et al., 2014; Liu et al., 2022). Closer parent–child relationships would enhance emotional support and family cohesion—both critical elements for fostering children’s social skills (Haven et al., 2014; Liu et al., 2022), creating opportunities for children to observe and internalize prosocial behaviors (Yamaoka and Bard, 2019; Zhong et al., 2020). Furthermore, children of parents with higher PCEs have been shown to exhibit fewer prosocial difficulties and fewer mental health concerns (Zhu et al., 2023; Blackwell et al., 2024). Since fewer prosocial difficulties in children are generally associated with higher children’s social skills, such as the demonstration of appropriate social behaviors and constructive coping strategies, it is reasonable to hypothesize that higher parental PCEs are positively associated with improved children’s social skills (Eisenberg et al., 1996).

Despite these insights, there remains limited research on the intergenerational transmission of parental PCEs. Two studies have shown that parental PCEs were associated with nurturing parenting attitudes (Morris et al., 2021) and improved family health (Daines et al., 2021), both of which are known contributors to children’s development. More direct evidence suggests that children with high parental PCEs scores exhibited fewer psycho-social problems and higher levels of well-being (Zhu et al., 2023; Blackwell et al., 2024). However, these studies primarily focus on broad health outcomes, leaving the relationship between parental PCEs and children’s social skills development largely unexplored.

### 1.2 The moderating role of the home-rearing environment

Parents’ childhood experiences are not decisive factors. Even parents with high PCEs may face barriers to translating these experiences into positive child outcomes. The home-rearing environment is central to understanding how parental positive childhood experiences (PCEs) influence children’s social skill development, as it constitutes the primary microsystem within Ecological Systems Theory (Bronfenbrenner, 2000). This theory posits that children’s development is shaped by interactions within and across multiple environmental systems, among which micro-systems have the most direct impact on children. In this context, the home-rearing environment serves as a critical micro-system, which interacts dynamically with parental characteristics and transforms into children’s social outcomes. As the most immediate setting, the home-rearing environment directly shapes children’s day-to-day experiences and serves as the primary context through which parental characteristics are transmitted to children.

The quality of the home-rearing environment, defined by factors such as emotional warmth, positive parent–child interactions, and avoidance of harsh discipline, can either amplify or diminish the intergenerational benefits of PCEs. Empirical evidence from Western indicates that family environments characterized by high emotional support and positive parent–child interactions strengthen the translation of parental PCEs into effective parenting behaviors (Schofield et al., 2014). Such environments provide children with frequent opportunities for social learning, which contributes to the development of emotional regulation and interpersonal skills (Feldman et al., 2023; Mirabile et al., 2018; Perry et al., 2020).

In contrast, the potential benefits of PCEs may be attenuated in less supportive home environments characterized by high stress, low emotional warmth, or culturally driven authoritarian practices. A US study demonstrated that family health mediates the relationship between parental positive childhood experiences (PCEs) and children’s adverse family experiences, revealing that unsupportive family environments attenuate the benefits of PCEs (Daines et al., 2021). Specifically, reduced socioemotional functioning and limited family resources are key characteristics of unsupportive home environments. In such contexts, the positive influence of parental PCEs on fostering a nurturing family system is weakened, which may increase developmental risks for children, including difficulties in social skills development. This is particularly relevant in the Chinese cultural context, where Confucian values often prioritize discipline, academic achievement, and parental authority over emotional expressiveness. For example, a study in China found that authoritarian parenting, prevalent in traditional Chinese families, restricted children’s autonomy, creativity, and emotional management (Ning, 2022; Li et al., 2023), even when parents had positive developmental histories. Consequently, the use of a Chinese sample is critical to explore these dynamics, as cultural factors uniquely shape parenting practices and home environments in China. The present study posits that the home-rearing environment moderates the association between parental PCEs and children’s social skills.

### 1.3 Current study

Empirical research examining the intergenerational effects of parental PCEs on the development of social skills in children remains limited, particularly within the Chinese sociocultural context. This study aims to examine the association between parental PCEs and preschool children’s social skills, and to assess whether home-rearing environment quality moderates this relationship.

## 2 Methods

### 2.1 Study design

This cross-sectional study was conducted in three kindergartens in Xuzhou, Shangqiu, and Tianjin, representing the eastern and northern regions of mainland China. Questionnaires were distributed to parents of preschool-aged children through kindergarten administrators, who provided informed consent forms and study information. Data were collected through multiple methods to assess main variables. Teachers conducted daily observations to evaluate children’s social skills, providing an objective measure based on classroom interactions. Concurrently, parents completed a self-reported questionnaire that included demographic information, details on parental positive childhood experiences (PCEs), and characteristics of the home-rearing environment.

The study included children aged 3–6 years residing with at least one parent. Children with disabilities, clinically diagnosed developmental delays, or parents with health conditions that could interfere with participation were excluded. Ethical approval was obtained from the University of Tsukuba (Approval No. 2035), and written informed consent was obtained from all participants.

### 2.2 Participants

To determine the appropriate sample size for detecting a potential moderating effect, *a priori* power analysis was conducted using G*Power 3.1 statistical software (Faul et al., 2007). Based on Cohen’s guidelines, a large effect size (f^2^ = 0.35) was assumed for the moderation analysis, with a significance level (*α*) of 0.05 and statistical power (1 − *β*) of 0.7 (Cohen, 1977). The analysis yielded a minimum required sample size of 102 participants.

A total of 491 questionnaires were distributed across the selected kindergartens. Following the application of inclusion and exclusion criteria, and the removal of questionnaires with incomplete data (missing demographic information or core outcome measures), a final sample of 320 completed questionnaires was retained for analysis. Details of the sampling strategy of the study and the flow chart of included participants are described in Supplementary Figure S1.

### 2.3 Measures

#### 2.3.1 Positive childhood experiences

Parental PCEs were assessed using the Benevolent Childhood Experiences (BCEs) Scale (Narayan et al., 2018), which has demonstrated acceptable validity and reliability in Chinese populations (Zhan et al., 2021). The scale was completed by parents, who retrospectively reported on their positive experiences prior to age 18. The BCEs Scale comprises 10 dichotomous (yes, no) items assessing dimensions such as predictable and positive quality of life, social support, and perceived safety. Each “yes” response is scored as 1, and each “no” response is scored as 0, with total scores ranging from 0 to 10. Higher scores indicate greater exposure to PCEs. In the current study, the scale showed satisfactory internal consistency (Cronbach’s *α* = 0.76).

#### 2.3.2 Social skills

Children’s social skills were assessed using the Social Skill Scale (SSS), an observation-based instrument scale with demonstrated validity and reliability for use with preschool-aged children (Anme et al., 2013). The SSS has shown cross-cultural applicability and measurement invariance in both Japanese and Chinese populations (Zhu et al., 2023). The SSS consists of 24 items across three subdomains: assertion, self-control and cooperation. Each item was scored on a 3-point Likert scale (0 = rarely, 1 = sometimes, 2 = always) according to daily observations in kindergartens by teachers. Subdomain scores are calculated as the sum of the respective items (range: 0–16 per subdomain), and the total score ranges from 0 to 48, with higher scores indicating greater social competence. In the present study, the SSS demonstrated excellent internal consistency (Cronbach’s *α* = 0.93).

#### 2.3.3 Home-rearing environment

The Index of Child Care Environment (ICCE) was used to assess the quality of the home-rearing environment, which is strongly correlated with the Home Observation for Measurement of the Environment (HOME) and has been widely used in Chinese research with robust reliability and validity (Zhu et al., 2023; Wang et al., 2024). The ICCE was completed by parents, who reported on their home environment and parenting practices. The scale includes 13 items across four dimensions: family interaction, external contact, avoidance of punishment, and social support. Total scores range from 0 to 13, with higher scores indicating more supportive and nurturing home-rearing environments. The ICCE demonstrated acceptable internal consistency in this study (Cronbach’s α = 0.81).

#### 2.3.4 Covariates

Covariates included parental relationship to the child, child’s age, child’s sex, sibling status, family structure, parental age, parental education background, and family income.

### 2.4 Statistical analysis

Quantitative data were analyzed using SPSS software version 26.0. Descriptive statistics and Pearson’s correlation analyses were conducted to analyze the study variables. First, Univariate regression of parental PCEs and home-rearing environment on children’s social skills. To test the moderating effect of the home-rearing environment on the association between parental PCEs and children’s social skills, a moderation analysis was conducted using PROCESS Macro (Model 1) for SPSS (Hayes, 2017). In this model, PCEs were entered as the independent variable, children’s social skills as the dependent variable, and the home-rearing environment as the moderating variable. Covariates included the child’s sex, child’s age, and other demographic variables. Bootstrapping with 5,000 resamples was used to estimate the 95% confidence intervals for the significance of effects.

## 3 Results

### 3.1 Descriptive characteristics of the sample

A total of 320 valid responses were included in the final analysis. Table 1 presents the demographic characteristics of the participants. The mean age of the children was 4.76 years (SD = 1.01). Among the children, 52.2% were male (n = 167) and 47.8% were female (n = 153). The primary caregiver was predominantly the mother (86.6%, n = 277), while 13.4% (*n* = 43) reported the father as the caregiver.

**Table 1:** Demographic characteristics (*n* = 320).

Variables	Categories	n	%
Gender	Male	167	52.2
	Female	153	47.8
Siblings	Yes	179	55.9
	No	141	44.1
Family structure	Single Parent	23	7.2
	Nuclear	208	65.0
	Extended	89	27.8
Parental relation to child	Father	43	13.4
	Mother	277	86.6
Parental age	<25y	2	0.6
	25-30y	43	13.4
	31-35y	148	46.3
	36-40y	92	28.7
	41-45y	32	10.0
	>45y	3	0.9
Parental education background	High School or Below	57	17.8
	Associate/Bachelor’s Degree	210	65.6
	Master’s Degree or Above	53	16.6
Family income	<100 thousand	69	21.6
	100–300 thousand	149	46.6
	300–500 thousand	50	15.6
	>500 thousand	52	16.2

### 3.2 Correlation analysis

Among the participating parents, 37.8% reported experiencing all 10 PCEs. The mean PCEs score was 0.83 (SD = 0.20). The mean score for the home-rearing environment was 0.78 (SD = 0.22), and the average social skills score was 1.50 (SD = 0.38). Table 2 presents the bivariate correlations among the primary study variables.

**Table 2:** Bivariate correlations among the main variables (*n* = 320).

	*M*	*SD*	Min	Max	1	2	3
1 PCEs	0.83	0.20	0.10	1.00	-		
2 Home-rearing environment	0.78	0.22	0.08	1.00	0.447^***^	-	
3 Social skills	1.50	0.38	0.42	2.00	0.564^***^	0.697^***^	-

^*^p < 0.05, ^**^p < 0.01, ^***^p < 0.001.

### 3.3 Moderation analysis

First, univariate models showed that parental PCEs were strongly associated with higher levels of children’s social skills (*β* = 1.15, *p* < 0.001), indicating that children of parents with more positive childhood experiences tend to exhibit greater social competence. Similarly, a more supportive home-rearing environment was associated with enhanced social skills in children (*β* = 1.14, *p* < 0.001), suggesting that more supportive families can foster stronger social abilities in preschoolers. A two-variable model (without interaction) yielded similar main effects (PCEs: *β* = 0.72, *p* < 0.001; home-rearing environment: *β* = 0.82, p < 0.001). The results are similar for uncontrolled for covariates (see Supplementary Tables S1, S2).

In the full moderation model (Table 3), the interaction term between PCEs and the home-rearing environment significantly predicted children’s social skills (*β* = 0.54, *p* = 0.027), indicating that the home-rearing environment significantly moderated the relationship between PCEs and children’s social skills. Unadjusted models (excluding covariates; see Supplementary Tables S3) yielded similar patterns.

**Table 3:** Moderating effect of home-rearing environment.

Relationship	Estimate	SE	t	95% CI	
				Lower	Upper
Constant	1.154	0.082	14.001^***^	0.992	1.317
PCEs → Social skills	0.801	0.077	10.357^***^	0.649	0.953
HRE → Social skills	0.851	0.065	13.163^***^	0.724	0.979
PCEs*HRE → Social skills	0.541	0.243	2.227^*^	0.063	1.018
Child’s sex → Social skills	0.008	0.024	0.329	−0.039	0.054
Child’s age → Social skills	0.048	0.012	3.839^***^	0.023	0.072
Siblings → Social skills	−0.006	0.024	−0.234	−0.052	0.041
Family structure → Social skills	−0.001	0.021	−0.053	−0.043	0.04
Relationship → Social skills	0.021	0.034	0.618	−0.046	0.089
Parental age → Social skills	0.009	0.013	0.699	−0.017	0.035
Parental education background → Social skills	0.034	0.021	1.593	−0.008	0.076
Family income → Social skills	0.006	0.003	2.146^*^	0.000	0.011

^*^p < 0.05, ^**^p < 0.01, ^***^p < 0.001. SS, social skills; HRE, home-rearing environment.

**Figure 1:**
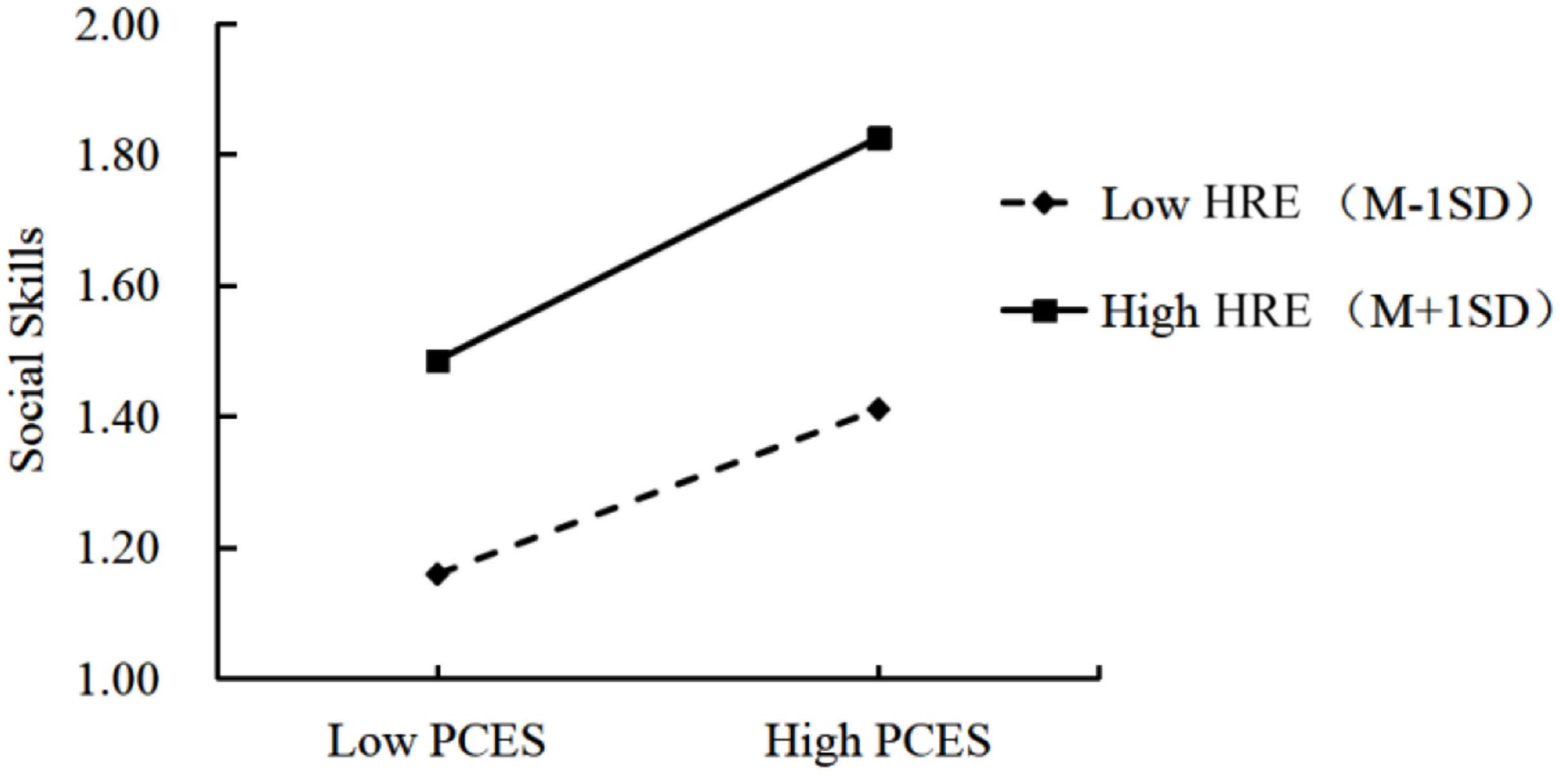
Potential patterns of parental positive childhood experiences, home-rearing environment and children’s social skills in moderation analysis model (*n* = 320).

To further interpret this interaction, a simple slope analysis was conducted (Figure 1). Results showed that when the home-rearing environment was at a low level (M - 1 SD), the positive association between PCEs and children’s social skills was weaker (simple slope = 0.68, *t* = 9.60, *p <* 0.01). Conversely, when the home-rearing environment was at a high level (M + 1 SD), the association was stronger (simple slope = 0.92, *t* = 8.372, *p* < 0.01). These findings demonstrate that the home-rearing environment significantly moderates the relationship between PCEs and children’s social skills.

## 4 Discussion

This study systematically examined the intergenerational influence of parental PCEs on children’s social skills, with particular emphasis on the moderating role of the home-rearing environment within the context of Chinese parenting culture. The findings indicate that parental PCEs are positively associated with children’s social skills, and that this relationship is significantly moderated by the home-rearing environment.

The observed association between parental PCEs and children’s social skills may be explained by the intergenerational transmission of parenting behaviors (Putallaz et al., 1998). Prior research demonstrates that maternal PCEs mitigate the adverse effects of ACEs on offspring prosocial deficits, underscoring PCEs as a pivotal determinant of children’s prosocial development (Zhu et al., 2023). This linkage arises from the profound influence of parental childhood experiences on subsequent parenting practices (Weiqiang et al., 2022). Parental with adverse childhood experiences is prone to overindulgent, permissive, authoritarian, or inconsistent styles, which engender emotional, behavioral, and peer-related difficulties in school-aged children (Yu et al., 2022). Conversely, parents with elevated PCEs exhibit positive parenting characterized by diminished psychological and physical aggression, enhanced mind-minded commentary, and improved parent–child interactions (Han et al., 2023), thereby attenuating child aggression, augmenting cooperation and conflict resolution capacities, and bolstering peer relational competencies (Stormshak et al., 2000; Parvin, 2016). Moreover, these parents serve as prosocial role models through their words, actions, and values, fostering children’s social and emotional abilities (Li et al., 2023).

Importantly, the current study offers novel insights into how the home-rearing environment moderates the relationship between parental PCEs and children’s social skills, emphasizing the critical role of family context in the intergenerational transmission of social competencies. According to Social Learning Theory, supportive home contexts marked by emotionally sensitive communication, diverse social experiences, and well-structured interactions provide abundant opportunities for modeling and timely reinforcement, thereby accelerating the internalization of children’s prosocial behaviors (Grusec and Lytton, 1988). Conversely, home environments characterized by low emotional warmth or high authoritarian control restrict meaningful parent–child exchanges, weakening the observational learning loops essential for social-skill acquisition (Shean, 2004; Vieira and Kraemer, 2022). Even parents with rich PCEs may struggle to transmit positive models if interactions are strained or punitive (Wolf et al., 2021). By contrast, when parents with high PCEs operate within nurturing, low-stress households, they can more reliably display responsive behaviors, which children readily emulate, fostering secure attachment and greater social confidence (Belsky, 1984; Koehn and Kerns, 2018; Zhang et al., 2019). Therefore, improving the quality of the home-rearing environment may amplify PCEs’ developmental benefits on children’s social skills.

In the Chinese context, however, Confucian values emphasizing discipline and authority often lead to authoritarian parenting, which may constrain opportunities for positive social interactions (Xie et al., 2022; Huang et al., 2024). Traditional Chinese parenting practices emphasize discipline, obedience, and structured behavioral guidance. While such practices may promote social order and responsibility, they may limit emotional expressiveness and parental warmth (Chao, 1994). Our findings suggest that when parents successfully balance warmth and structure—integrating emotional support with behavioral expectations—children are more likely to develop social confidence and effective social skills (Chen and French, 2008; Liu et al., 2022).

The study also indicated significant associations between key covariates—particularly children’s age and family income—and social skills. These findings are consistent with established developmental principles that identify early childhood, particularly the kindergarten period, as a crucial stage for social skill acquisition (Zhu et al., 2021). As language and cognition mature, children become more adept at navigating social interactions. Furthermore, children from families with higher income levels exhibited stronger social skills (Griffith et al., 2016; Davis and Qi, 2020). This disparity may be attributed to the greater financial resources available to higher-income families, enabling them to provide enriched educational opportunities, socially stimulating environments, and diverse extracurricular activities, which collectively enhance children’s social competence (Garratt et al., 2017).

The findings of this study are consistent with ecological systems theory, which emphasizes that children’s social development is influenced by a combination of individual factors (e.g., age), family factors (e.g., PCEs, home-rearing environment), and socioeconomic factors (e.g., family income) (Bronfenbrenner, 2000). Practically, this suggests that interventions should not only address parental well-being but also target the home environment. Parenting programs that foster emotionally supportive, cognitively stimulating, and structured interactions may enable parents to draw more effectively on their positive childhood experiences when nurturing their children’s social skills.

Despite its contributions, this study has several limitations. First, the study relies primarily on parent self-reported measures (e.g., PCEs, home-rearing environment), which may introduce biases like social desirability and recall inaccuracies. While SSS used teacher observations, future research should add multi-informant or direct observational methods for all measures to enhance validity. Second, the study sample was limited to preschool-aged children from three urban areas in China, with the majority in middle to upper preschool grades (ages 4–5). The urban sample from three cities limits generalizability to rural or diverse socioeconomic contexts in China. Future studies should include more diverse samples across various regions and developmental stages. Finally, this study cannot establish causality because it used a cross-sectional design. Future research should use a longitudinal design to determine whether the effects of parental positive childhood experiences on children’s social skills are moderated by the home-rearing environment.

## 5 Conclusion

The study revealed two key findings. First, parental PCEs showed a significant positive association with children’s social skills. Second, the home-rearing environment played a moderating role, with parental PCEs demonstrating a stronger influence on children’s social skills in more supportive home-rearing environments. These findings underscore the need to consider both parents’ early experiences and current family contexts when understanding and promoting early social development in children.

## Data Availability

The original contributions presented in the study are included in the article/Supplementary material, further inquiries can be directed to the corresponding author.
