# Supplementary material for: Intergenerational effects of parental positive childhood experiences on social skills development in Chinese preschoolers: the moderating role of the home-rearing environment
Source: Front Psychol. 2025 Nov 10;16:1679531. doi: 10.3389/fpsyg.2025.1679531 (PMC12646171; doi:10.3389/fpsyg.2025.1679531)
Supplement: Supplementary file 1 [file Table_1.docx]

**Figure S1 Flow Diagram of Included Participants**

491 children

(Aged 3-6 years old)

412 children

Refused to participate n=79

(Response rate: 84.0%)

320 children

Missing data:

Parental PCEs (n = 18)

Social Skills (n = 34)

Home-rearing Environment (n = 26)

Demographic variables (n = 14)

Table S1 Univariate regressions for parental PCEs and home-rearing environment

|  | Model 1^a^ | | Model 2^b^ | |
| --- | --- | --- | --- | --- |
|  | *β* (95% CI) | P value | *β* (95% CI) | P value |
| PCEs | 1.29 (1.15, 1.44) | **0.000** | 1.15 (1.01, 1.30) | **0.000** |
| HRE | 1.27 (1.14, 1.40) | **0.000** | 1.14 (1.02, 1.27) | **0.000** |

Note. HRE = home-rearing environment.

^A^ Model 1:No adjust.

^B^ Model 2:Adjusted for child's sex, child's age, sibling, family structure, parental relationship to the child, parental age, parental educational background, and family income

Table S2 Two-variable regressions (without interaction) for parental PCEs and home-rearing environment

|  | Model 1^a^ | | Model 2^b^ | |
| --- | --- | --- | --- | --- |
|  | *β* (95% CI) | P value | *β* (95% CI) | P value |
| PCEs | 0.79 (0.65, 0.92) | **0.000** | 0.72 (0.59, 0.86) | **0.000** |
| HRE | 0.88 (0.76, 1.01) | **0.000** | 0.82 (0.70, 0.95) | **0.000** |

Note. HRE = home-rearing environment.

^A^ Model 1:No adjust.

^B^ Model 2:Adjusted for child's sex, child's age, sibling, family structure, parental relationship to the child, parental age, parental educational background, and family income

Table S3 Two-variable regressions (with interaction) for parental PCEs and home-rearing environment

|  | Model 1^a^ | | Model 2^b^ | |
| --- | --- | --- | --- | --- |
|  | *β* (95% CI) | P value | *β* (95% CI) | P value |
| PCEs | 0.87 (0.72, 1.02) | 0.000 | 0.80 (0.65, 0.95) | 0.000 |
| HRE | 0.92 (0.79, 1.04) | 0.000 | 0.85 (0.72, 0.98) | 0.000 |
| PCEs*HRE | 0.62 (0.14, 1.10) | **0.012** | 0.54 (0.06, 1.02) | **0.027** |

Note. HRE = home-rearing environment.

^A^ Model 1:No adjust.

^B^ Model 2:Adjusted for child's sex, child's age, sibling, family structure, parental relationship to the child, parental age, parental educational background, and family income
